# On Transform Domain Communication Systems under Spectrum Sensing Mismatch: A Deterministic Analysis

**DOI:** 10.3390/s17071594

**Published:** 2017-07-08

**Authors:** Chuanxue Jin, Su Hu, Yixuan Huang, Qu Luo, Dan Huang, Yi Li, Yuan Gao, Shaochi Cheng

**Affiliations:** 1 National Key Lab on Communication, University of Electronic Science and Technology of China, 611731 Chengdu, China; jincx@uestc.edu.cn (C.J.); husu@uestc.edu.cn (S.H.); 201521260211@std.uestc.edu.cn (Y.H.); 201621260210@std.uestc.edu.cn (Q.L.); huangdan@uestc.edu.cn (D.H.); 2 The High School Affiliated to Renmin University of China, 100080 Beijing, China; liyi@rdfz.cn; 3 China Defense Science and Technology Information Center, 100048 Beijing, China; success.no.1@163.com; 4 Department of Electronic Engineering, Tsinghua University, 100084 Beijing, China

**Keywords:** transform domain communication systems, spectrum sensing mismatch, cyclic code shift keying, internet of things

## Abstract

Towards the era of mobile Internet and the Internet of Things (IoT), numerous sensors and devices are being introduced and interconnected. To support such an amount of data traffic, traditional wireless communication technologies are facing challenges both in terms of the increasing shortage of spectrum resources and massive multiple access. The transform-domain communication system (TDCS) is considered as an alternative multiple access system, where 5G and mobile IoT are mainly focused. However, previous studies about TDCS are under the assumption that the transceiver has the global spectrum information, without the consideration of spectrum sensing mismatch (SSM). In this paper, we present the deterministic analysis of TDCS systems under arbitrary given spectrum sensing scenarios, especially the influence of the SSM pattern to the signal to noise ratio (SNR) performance. Simulation results show that arbitrary SSM pattern can lead to inferior bit error rate (BER) performance.

## 1. Introduction

The fifth generation (5G) of wireless systems is designed to fuel new communication paradigms, such as the Internet of Things (IoT) and mobile Internet services, where numerous sensors and devices are interconnected and demanded for improvements of several issues. The rapid development of IoT has triggereda 1000-fold data traffic increase by 2020 for 5G and related network scenarios [1]. However, it introduces a large amount of unexpected electromagnetic interference that may cause the failure of the transmission. Therefore, investigating higher spectral efficiency technology becomes one of the key breakthroughs. Meanwhile, due to the fast growth of IoT, 5G also needs to support massive access among users and/or devices [2,3].

To achieve higher transmission speed and more reliable QoS (quality of services), future wireless communication systems require more smart waveforms to tackle frequency band scarcity and various interferences. By introducing the concept of cognitive radio (CR) [4,5,6,7], wireless networks could obtain better frequency utilization and transmission, leading to more robust transmission. To accommodate the rapidly increasing demand for wireless broadband communications in smart grid networks, research efforts are currently ongoing to enable the networks to utilize the TV spectrum according to the cognitive radio paradigm [8,9]. In recent years, regulatory bodies, such as the FCC, have approved the dynamic access of unlicensed sensor networks, referred to in the following as secondary sensor networks to the TV white space (TVWS) spectrum [10,11,12]. The existing regulations circumvent the need for sensing algorithms for establishing the availability of free TVWS spectrum [13,14,15]. In [16], the authors presented a systematic approach to exploit TVWS for device-to-device communications with the aid of the existing cellular infrastructure. In TVWS, the unlicensed users are required to periodically access a database so as to acquire information on the spectrum usage of the licensed users. The authors in [17] developed a stochastic analytical framework that allows us to account for the PU activity dynamics, the quality dynamics among the different channels, and the overhead induced by the database access. In [18], the TVWS authors studied the throughput achievable by an unlicensed sensor network over the TV white space spectrum in the presence of coexisting interference. Detecting the signals of primary users in the wideband spectrum is a key issue for cognitive radio networks [19]. In [20], the authors proposed a novel signal detection algorithm based on the Riemannian distance and Riemannian mean, which is different from the traditional eigenvalue-based detector derived with the generalized likelihood ratio criterion.

Following a general analytic framework, named spectrally-modulated and spectrally-encoded [21,22,23], various multicarrier waveforms can be generated based on the need of CR user, e.g., orthogonal frequency division multiplex (OFDM) and multicarrier code division multiple access (MC-CDMA) [24]. As another type of overlay CR application, the transform domain communication system (TDCS) was proposed for supporting reliable communications with low spectral density through spectrum bin nulling and frequency domain spreading [25]. Hence, TDCS can be viewed as a proactive anti-jammer transmission scheme when some spectrum bands are disturbed by intentional jammers [26]. The foundations of TDCS are established by the Air Force Institute of Technology [27,28], which is able to dynamically update systematic parameters using spectrum sensing and spectrum nulling. To simplify the implementation complexity of TDCS, the authors in [29] proposed an efficient OFDM-based TDCS transceiver. Recently, by analyzing the advantages of sequence investigation and a two-dimension spreading method, a series of advances have been presented to improve TDCS for practical utilizations [7,8], i.e., peak to average power ratio problems, ideal multi-user networks without interference, etc.

Especially, some of the ideas have attempted to tackle the situation of spectrum sensing mismatch (SSM). However, the concept of SSM is simple, using direct statistical observation, and neither explicit mathematical models nor systematic deduction has been ever presented. Thus, the deterministic analysis of TDCSs under SSM is still an open problem. In this paper, we focus on the performance metric introduced by SSM on TDCS systems in terms of bit error ratio (BER). As per theoretical analysis, two different kinds of modulation schemes of TDCS are given, called cyclic code shift keying (CCSK) and quadrature phase shift keying (QPSK). CCSK is widely adopted in TDCS because of its low probability of interception [25], while QPSK is usually adopted as an embedded symbol to enhance the spectral efficiency [30]. A general definition is provided to measure the level of SSM. According to the theoretical analysis and simulation results, the influence of SSM can be modeled as the product of coefficient multiplied and the traditional signal to noise ratio (SNR). The expression of the mismatch factor, which only depends on the spectrum sensing results, can be easily modeled.

The rest of this paper is organized as follows: In Section 2, we simply introduce two types of TDCS systems with different modulation methods. Then, the definition of SSM is given in Section 3, and the theoretical analysis of BER performance is given to provide the convinced evaluation for the influence of SSM. Finally, comprehensive simulations and analysis are presented in Section 4 and conclusions are given in Section 5.

## 2. System Models and Modulation Schemes

### 2.1. Transmitter Side of the TDCS

**Figure 1:**
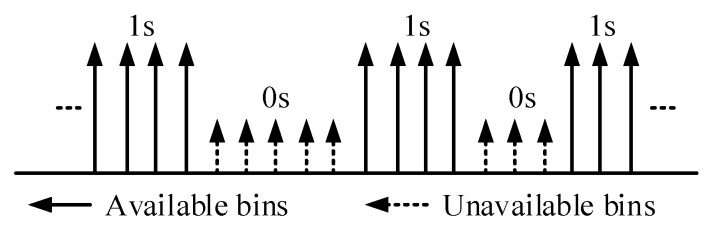
An example of the spectrum sensing result for vector A.

Spectrum sensing is recognized as the first step in the TDCS. The whole spectrum band is divided into *N* spectral bins when adopting the TDCS. A spectrum mask vector is used to tag the availability of such spectral bins with $A=\{A_{0},A_{1},...,A_{N-1}\}$ . The value $A_{k}$ is set to 1 (or 0) if the magnitude is smaller (or larger) than the given threshold, as shown in Figure 1. It is often considered that all the spectral bins form a mathematical set $\Omega^{C}$ , where $\left\|\Omega^{C}\right\|=N_{C}$ denotes the number of spectral bins that are not occupied, i.e., $\left\{A_{k}=1,k\in \Omega^{C}\right\}$ . To form the waveform, like noise, a user-specific pseudorandom (PR) poly-phase vector, i.e., $P=\{e^{jm_{0}},e^{jm_{1}},...,e^{jm_{N-1}}\}$ , is generated by a phase mapping process [23]:

(1) $$m_{k}\in \left\{0,\frac{2\pi }{2^{r}},\frac{4\pi }{2^{r}},...,\frac{2\pi \left(2^{r}-1\right)}{2^{r}}\right\}$$

**Figure 2:**
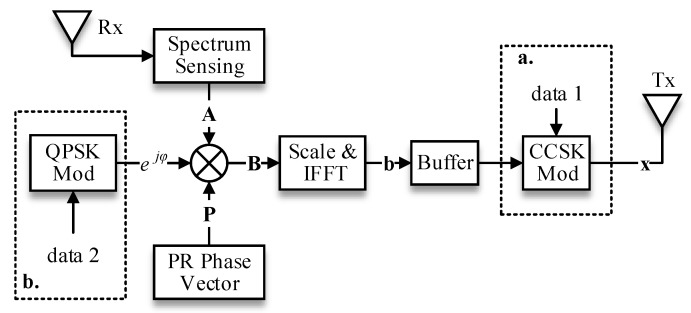
Block diagram of the TDCS transmitter (two modulation methods in one general diagram).

On the transmitter side, illustrated in Figure 2, the pseudorandom poly-phase vector **P** is multiplied one-by-one with the spectrum availability vector A to generate a spectral vector, i.e., $B=\left\{B_{0},B_{1},...,B_{N-1}\right\}=A\cdot P$ . A fundamental modulation waveform (FMW) b is yielded by performing an IFFT operation on this spectral vector B as follows:

(2) $$\begin{array}{l} b=\left\{b_{0},b_{1},...,b_{N-1}\right\}=\lambda F^{-1}\left(B\right)\\ b_{n}=\lambda \frac{1}{\sqrt{N}}\sum_{k\in \Omega^{C}}\left(e^{jm_{k}}\right)e^{j2\pi kn/N} \end{array}$$

where $\lambda =\sqrt{N/N_{C}}$ denotes a scaling factor that ensures the normalized transmission power. To this level, the generation of the smart waveform for dynamic spectrum utilization is ready and cached in a buffer for later modulation processing.

### 2.2. Two Waveform Modulation Schemes

In a typical CCSK, the cyclic-shifted sequence (namely the FMW b in the TDCS) is used to compose a data symbol. For *M*-ary CCSK signaling, $\log_{2}\left(M\right)$ bits of data are gathered to map a complete data symbol, i.e., $S_{C}\in \left\{0,1,...,M\right\}$ (data1 in Figure 2), referring to any given alphabet (we assume M=N in this work for convenience). When an *M*-ary order CCSK is used, the signal to be transmitted is generated by cyclically shifting by τ∈{0,1,...,M} :

(3) $${\left(x\right)}^{\left(CCSK\right)}=\left\{x_{0},x_{1},...,x_{N-1}\right\}=\left\{b_{\tau },b_{\tau +1},...,b_{N},b_{0},...,b_{\tau -1}\right\}$$

According to Han’s OFDM-based implementation [25], the CCSK operation can be replaced by using FFT to reduce computational complexity:

(4) $${\left(x_{n}\right)}^{\left(CCSK\right)}=\lambda \frac{1}{\sqrt{N}}\sum_{k\in \Omega^{C}}\left(e^{jm_{k}}e^{-j2\pi kS_{C}/N}\right)e^{j2\pi kn/N}$$

Compared the CCSK-based TDCS, the QPSK-based TDCS removes the cyclic-shift operation and acts more like a multi-carrier CDMA (MC-CDMA). Before IFFT operation, a given QPSK symbol, $S_{Q}=e^{j\varphi }$ (data2 in Figure 2), is spread directly over each subcarrier of the spectral waveform **B**, as depicted in Figure 2. The corresponding transmitted signal is given as:

(5) $${\left(x_{n}\right)}^{\left(QPSK\right)}=\lambda \frac{1}{\sqrt{N}}\sum_{k\in \Omega^{C}}\left(e^{jm_{k}}e^{j\varphi }\right)e^{j2\pi kn/N}$$

Comparing [26] with [25], different modulations result in different transmitting waveforms. Despite different modulation methods are employed, the above-mentioned two TDCS models are fully compatible with OFDM transmission due to its multicarrier structure, as mentioned in [27].

### 2.3. Receiver Side of the TDCS

**Figure 3:**
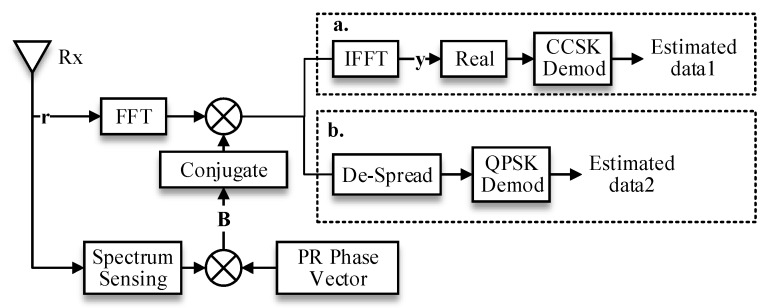
Block diagram of the TDCS receiver (two modulation methods in one general diagram).

**Figure 4:**
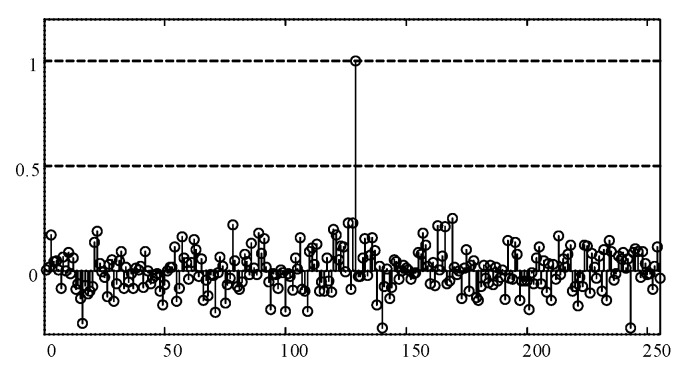
An example of a PCF vector (with noise).

Traditionally, it is assumed that the TDCS receiver shares the same spectrum sensing result with the transmitter in order to simplify the analysis. The TDCS receiver is depicted in Figure 3 in a general diagram for both modulations. We assume the received signal is $r=\left\{r_{0},r_{1},...,r_{N-1}\right\}$ for both models. For CCSK signaling, this received signal is multiplied element-by-element with a replica of the spectral waveform B to generate a periodic cyclic function (PCF):

(6) $$y=\left\{y_{0},y_{1},...,y_{N-1}\right\}=F^{-1}\left\{\left(F\left(r\right)\right)\cdot {\left(B\right)}^{*}\right\}$$

where ${\left(\cdot \right)}^{*}$ denotes complex conjugate operation. Note that this PCF waveform should be like an impulse response shape [25] as depicted in Figure 4:

(7) $$y_{\tau }=\lambda \sum_{k\in \Omega^{C}}e^{{\langle j2\pi k\left(\tau -S_{C}\right)/N\rangle }_{N}}\overset{IFFT}{\underset{FFT}{↔}}\delta \left({\langle \tau -S_{C}\rangle }_{N}\right)$$

where ${\langle m\rangle }_{N}=m\mathrm{mod}N$ , and δ(τ) is the impulse response function. Thus, the estimated CCSK symbol is recovered by detecting the index of the maximum value in PCF vector (only the real part is considered):

(8) $${\left({\hat{S}}_{C}\right)}^{\left(CCSK\right)}=\underset{\tau }{\underset{︸}{\arg\max}}\left\{ℜ\left(y_{\tau }\right)\right\}$$

where ℜ(⋅) denotes the operation of obtaining the real value of a complex quantity.

In the QPSK-TDCS model, the operations of the receiver are pretty much the same except that one QPSK symbol message is spreaded over every spectral bin, and a conventional de-spread operation is needed to recover the estimated QPSK symbol:

(9) $${\left({\hat{S}}_{Q}\right)}^{\left(QPSK\right)}=\frac{1}{N}\sum \left\{\left(F\left(r\right)\right)\cdot {\left(B\right)}^{*}\right\}$$

## 3. Spectrum Sensing Mismatch

### 3.1. Mathematical Model for Spectrum Sensing Mismatch

**Figure 5:**
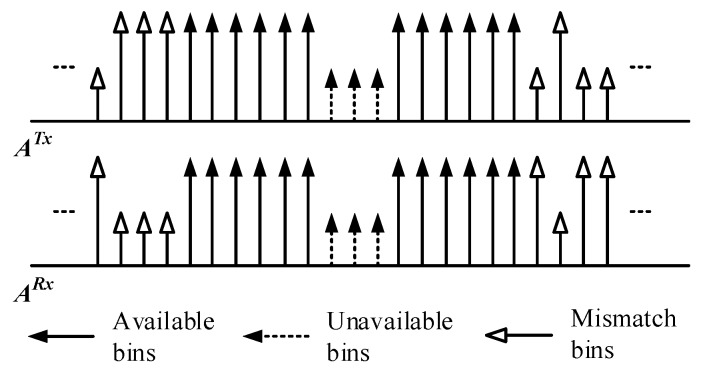
An example of spectrum sensing mismatch.

In wireless systems, the transceivers are often deployed separately from each other and additional interferences will be involved during such a process, which is even severe in IoT systems. It has been defined in IEEE802.22 [29] that channel uncertainties are likely to introduce a mismatch between the spectrum sensing results. Atypical example is depicted in Figure 5, which is named Spectrum Sensing Mismatch (SSM) in this work, where spectrum sensing results forany certain spectral bins are different in the transceiver.

In these available spectral bins, there are total three of them in the presence of SSM:

-
$\left\{A_{k}=1,k\in \Omega^{Tx}\right\}$ for those bins available only at the transmitter side, with $\left\|\Omega^{Tx}\right\|=N_{Tx}$ ;
-
$\left\{A_{k}=1,k\in \Omega^{Rx}\right\}$ for those bins available only at the transmitter side, with $\left\|\Omega^{Rx}\right\|=N_{Rx}$ ; and
-
$\left\{A_{k}=1,k\in \Omega^{C}\right\}$ for those bins available at the same time both at the transmitter and the receiver, with $\left\|\Omega^{C}\right\|=N_{C}$ .

For any spectrum sensing results, it satisfies:

(10) $$\left\{ \begin{array}{l} \Omega^{C}=\Omega^{Tx}\cap \Omega^{Rx}\\ 0\leq N_{C}\leq \left(N_{Tx},N_{Rx}\right)\leq N \end{array}\right.$$

**Figure 6:**
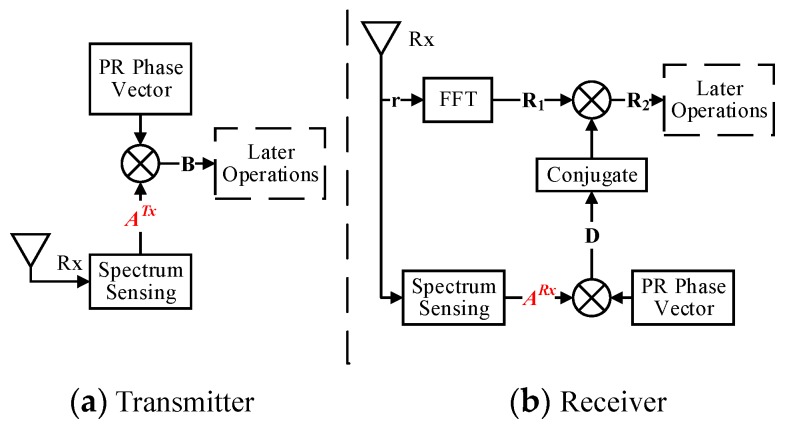
Simplified block diagram of TDCS transceivers.

When the TDCS is essentially a multi-carrier system, the mismatched spectral bins will significantly lead to the loss of signal power. The Simplified block diagram of TDCS transceivers is depicted in Figure 6. Therefore, we have:

-
$D=A^{Rx}\cdot P$ for receiver’s local reference signal, where $\left\{A_{k}^{Rx}=1,k\in \Omega^{Rx}\right\}$ ;
-
$R_{1}=F\left(r\right)$ for the received signal in the frequency domain (after equalization);
-
$R_{2}=R_{1}\cdot {\left(D\right)}^{*}$ for those after multiply with local reference signal $R_{1}$ ; and
-
α for the proposed mismatch factor, ( 0≤α≤1 ).

### 3.2. Deterministic Analysis of Performance in AWGN Channels

In this discussion, it is assumed that most of the signal processing operations for TDCS remains the same, and the attention is focused on the mismatch of spectral bins. For the CCSK model, substituting the introduced $\Omega^{Tx}$ for $\Omega^{C}$ in [17] generates:

(11) $${\left(x_{n}\right)}^{\left(CCSK\right)}=\lambda \frac{1}{\sqrt{N}}\sum_{k\in \Omega^{Tx}}\left(e^{jm_{k}}e^{-j2\pi kS_{C}/N}\right)e^{j2\pi kn/N}$$

After passing though the AWGN channel, the received signal $r=\left\{r_{0},r_{1},...,r_{N-1}\right\}$ can be expressed as:

(12) $${\left(r_{n}\right)}^{\left(CCSK\right)}=\lambda \frac{1}{\sqrt{N}}\sum_{k\in \Omega^{Tx}}\left(e^{jm_{k}}e^{-j2\pi kS_{C}/N}\right)e^{j2\pi kn/N}+w\left(n\right)$$

where w(n) denotes the additive white Gaussian noise with $E\left\{\left(w\left(n\right)\right){\left(w\left(n\right)\right)}^{H}\right\}=N_{0}$ . Thus, the frequency domain term is given by:

(13) $$\begin{array}{l} R_{1}\left(k\right)\left\{ \begin{array}{l} \lambda e^{jm_{k}}e^{-j2\pi kS_{C}/N}+n_{1}\left(k\right),\;k\in \Omega^{Tx}\\ n_{1}\left(k\right),\;otherwise \end{array}\right.\\ n_{1}\left(k\right)=\frac{1}{\sqrt{N}}\sum_{k=0}^{N}w\left(n\right)e^{j2\pi kn/N} \end{array}$$

The second term, $n_{1}\left(k\right)$ , denotes the output of the Gaussian noise after FFT operation. According to Parseval’s theorem and the property of independent Gaussian variables [18], it still maintains a Gaussian distribution. After multiplying with the local reference signal in an element-wise manner, we obtain:

(14) $$R_{2}(k)=\overset{signal}{\overset{︷}{R_{CCSK}(k)}}+\overset{noise}{\overset{︷}{n_{2}(k)}}$$

To be specific:

(15) $$\begin{array}{cc} R_{CCSK}\left(k\right) & =\left\{ \begin{array}{l} \lambda e^{-j2\pi kS_{C}/N},\;k\in \Omega^{C}\\ 0,\;otherwise \end{array}\right.\\ n_{2}\left(k\right) & =\left\{ \begin{array}{l} e^{-jm_{k}}\cdot n_{1}\left(k\right),\;k\in \Omega^{Rx}\\ 0,\;otherwise \end{array}\right. \end{array}$$

Notice that the difference of spectral bin index *k* in [16] between the signal term and noise term. Meanwhile, $n_{2}\left(k\right)$ still maintains a Gaussian noise when it is valid. Thus, the related PCF vector is generated after an IFFT operation, $y=F^{-1}\left(R_{2}\right)$ , with:

(16) $$y_{\tau }=\frac{1}{\sqrt{N}}\sum_{k=0}^{N-1}\left(R_{2}\left(k\right)\right)e^{j2\pi k\tau /N}=\lambda \cdot R{\langle \tau -S_{C}\rangle }_{N}+n_{3}\left(\tau \right)$$

where:

(17) $$\begin{array}{cc} R{\langle \tau \rangle }_{N} & =\frac{1}{\sqrt{N}}\sum_{k\in \Omega^{C}}e^{-j2\pi k\left(\tau \right)/N}\overset{IFFT}{\underset{FFT}{↔}}\delta \left({\langle \tau \rangle }_{N}\right)\\ n_{3}\left(\tau \right) & =\frac{1}{\sqrt{N}}\sum_{k\in \Omega^{Rx}}\left(n_{3}\left(k\right)\right)e^{j2\pi k\tau /N} \end{array}$$

Using the same CCSK detection as in [15], the estimated CCSK symbol is recovered. According to the property of IFFT/FFT, $R{\langle \tau \rangle }_{N}$ is a perfect impulse response signal providing all the spectrum bins available. However, due to the insufficiency of valid spectrum bins in the presence of SSM, the impulse response shape of $R{\langle \tau \rangle }_{N}$ is destroyed and its main peak is now down to $\left(N_{C}/N\right)$ . Thus, the SNR for CCSK-based TDCS under SSM is given as:

(18) (SNR)(CCSK)=(λNCN)212NRxNN0=(1−α)⋅(2εSN0)︸traditionalCCSK SNR

where:

(19) $$\alpha =1-\frac{{\left(N_{C}\right)}^{2}}{N_{Tx}N_{Rx}}$$

denotes the proposed mismatch factor as to measure the degree of SSM. Based on this, the bit error probability for the TDCS using the CCSK method is given using the well-accepted BER expression for *M*-ary orthogonal signaling [16]:

(20) $$\left(P_{b}\right)=\frac{2^{\log_{2}\left(M\right)-1}}{2^{\log_{2}\left(M\right)}-1}\cdot \frac{1}{\sqrt{2}}\cdot \int_{-\infty }^{+\infty }\left[1-{\left(\frac{1}{\sqrt{2\pi }}Q\left(y\right)\right)}^{M}\right]\cdot \exp\left[-\frac{1}{2}{\left(y-\sqrt{\left(1-\alpha \right)\left(2\frac{\varepsilon_{S}}{N_{0}}\right)}\right)}^{2}\right]dy$$

where $Q\left(y\right)=\left(1/\sqrt{2\pi }\right)\int_{y}^{\infty }\exp\left(-u^{2}/2\right)du$ denotes the error function. Obviously a larger value of α indicates a worse spectrum match pattern, leading to an inferior BER performance.

For the QPSK-based TDCS model, the transmitted signal remains the same as in (5), except that $k\in \Omega^{Tx}$ and $\lambda =\sqrt{N/N_{Tx}}$ . In the receiver, after multiply with a receiver reference waveform, the frequency domain signal, i.e., $R_{2}=R_{1}\cdot {\left(D\right)}^{*}=\left\{R_{2}\left(0\right),R_{2}\left(1\right),...,R_{2}\left(N-1\right)\right\}$ , is:

(21) $$R_{2}(k)=\overset{signal}{\overset{︷}{R_{QPSK}(k)}}+\overset{noise}{\overset{︷}{n_{2}(k)}}$$

where $R_{QPSK}\left(k\right)=\lambda e^{j\varphi }$ is valid only when $k\in \Omega^{C}$ , and $n_{2}\left(k\right)$ is of the same expression as in [16]. After de-spreading, the estimated QPSK symbol is given by:

(22) $${\hat{S}}_{Q}=\frac{1}{N}\sum_{k=0}^{N-1}R_{2}\left(k\right)=\frac{N_{C}}{N}\lambda e^{j\varphi }+n_{3}$$

(23) $${\left(SNR\right)}^{\left(QPSK\right)}=\frac{{\left(\frac{N_{C}}{N}\lambda e^{j\varphi }\right)}^{2}}{\frac{N_{Rx}}{N^{2}}N_{0}}=\left(1-\alpha \right)\underset{\begin{array}{l} traditional\\ QPSK\;SNR \end{array}}{\underset{︸}{\left(\frac{\varepsilon_{S}}{N_{0}}\right)}}$$

Similarly, $\alpha =\left(1-{\left(N_{C}\right)}^{2}/N_{Tx}N_{Rx}\right)$ remains the same as in (22) despite a different modulation method is employed. Following the typical SNR-BER expression for QPSK [17], the related bit error performance is:

(24) $${\left(P_{b}\right)}^{\left(QPSK\right)}=2Q\left(\sqrt{\frac{2\varepsilon_{b}}{N_{0}}}\right)\left[1-\frac{1}{2}Q\left(\sqrt{\frac{2\varepsilon_{b}}{N_{0}}}\right)\right]$$

where $\varepsilon_{b}=\left(1/2\right)\varepsilon_{S}$ denotes average bit power for QPSK signaling.

### 3.3. Semi-Deterministic Analysis of Performance in Multipath Fading Channels

For multipath Rayleigh fading channels, a well-established discrete-time finite channel response is adopted as $h_{l}$ with l∈{0,1,...,L−1} (*L* is the number of total channel paths). Thus, the channel response in the frequency domain is given as:

(25) $$H_{k}=\sum_{l=0}^{L-1}h_{l}\cdot e^{-j2\pi kl/N},\;k=0,1,...,N-1$$

In this paper, a linear one-tap frequency-domain zero-forcing (ZF) equalizer, $G=\left\{G_{0},G_{1},...,G_{N-1}\right\}$ , is employed providing perfect channel estimation:

(26) $$G_{k}=\frac{1}{H_{k}},\;k=0,1,...,N-1$$

For the CCSK-based TDCS, after performing normal OFDM demodulation and equalization, the received signal in the frequency domain is given as:

(27) $$\begin{array}{l} R_{1}\left(k\right)\left\{ \begin{array}{l} \lambda e^{jm_{k}}e^{-j2\pi kS_{C}/N}+n_{1}\left(k\right),\;k\in \Omega^{Tx}\\ n_{1}\left(k\right),\;otherwise \end{array}\right.\\ n_{1}\left(k\right)=\frac{1}{H_{k}\cdot \sqrt{N}}\sum_{k=0}^{N}w\left(n\right)e^{j2\pi kn/N} \end{array}$$

Compared to Equation (26), the signal term remains the same. However, the noise term $n_{1}\left(k\right)$ is divided by the frequency channel response of *k*th subcarrier, resulting in the change of noise variance:

(28) $$n_{3}\left(\tau \right)=\frac{1}{\sqrt{N}}\sum_{k\in \Omega^{Rx}}\left(\frac{1}{H_{k}\cdot \sqrt{N}}\sum_{k=0}^{N}w\left(n\right)e^{j2\pi kn/N}e^{-jm_{k}}\right)e^{j2\pi k\tau /N}$$

Thus, the SNR for the CCSK-based TDCS under SSM in the multipath Rayleigh fading channel is written as:

(29) $${\left(SNR\right)}^{\left(CCSK\right)}=\frac{{\left(N_{C}\right)}^{2}}{N_{Tx}\left(\sum_{k\in \Omega^{Rx}}\left(1/{\left(H_{k}\right)}^{2}\right)\right)}\cdot \left(2\frac{\varepsilon_{S}}{N_{0}}\right)=\left(1-\alpha \right)\cdot \underset{\begin{array}{l} traditional\\ CCSK\;SNR \end{array}}{\underset{︸}{\left(2\frac{\varepsilon_{S}}{N_{0}}\right)}}$$

Following the concept of the mismatch factor α here, this value now becomes:

(30) $$\alpha =1-\frac{{\left(N_{C}\right)}^{2}}{N_{Tx}\left(\sum_{k\in \Omega^{Rx}}\left(1/{\left(H_{k}\right)}^{2}\right)\right)}$$

The BER performance can be easily derived using the same expression in [17]. For the QPSK-based TDCS, the performance analysis can be derived by following the same idea, and the final SNR under SSM is given as:

(31) $${\left(SNR\right)}^{\left(QPSK\right)}=\frac{{\left(N_{C}\right)}^{2}}{N_{Tx}\left(\sum_{k\in \Omega^{Rx}}\left(1/{\left(H_{k}\right)}^{2}\right)\right)}\cdot \left(\frac{\varepsilon_{S}}{N_{0}}\right)=\left(1-\alpha \right)\cdot \underset{\begin{array}{l} traditional\\ QPSK\;SNR \end{array}}{\underset{︸}{\left(\frac{\varepsilon_{S}}{N_{0}}\right)}}$$

Likely, it is found that α remains the same value as in Equation (30), and the BER performance expression still satisfies Equation (31).

From our analysis above, a mismatch factor with an explicit mathematical expression has been introduced to precisely measure the degree of spectrum sensing mismatch. It should be noticed that in the AWGN channel, the value of α only depends on the spectrum sensing result and the analysis is deterministic; whereas in the multipath Rayleigh fading channel, it is referred to as semi-deterministic analysis since perfect estimation of the channel response is required.

## 4. Simulation Results

In this part, the number of spectrum bins is set to *N* = 256. 256-ary CCSK modulation and QPSK modulation are introduced, respectively. Based on the above analysis, the mismatch factor is set to $\alpha ={\left(N_{C}\right)}^{2}/N_{Tx}N_{Rx}$ . Meanwhile, a random spectral bins availability method is assumed to obtain a better BER performance. For the multipath Rayleigh fading channel, a typical COST207RAx6 channel is used, the Mismatch Factor is shown in Table 1, which are used as simulation parameters. More details are available in [18].

### 4.1. AWGN Channels

**Figure 7:**
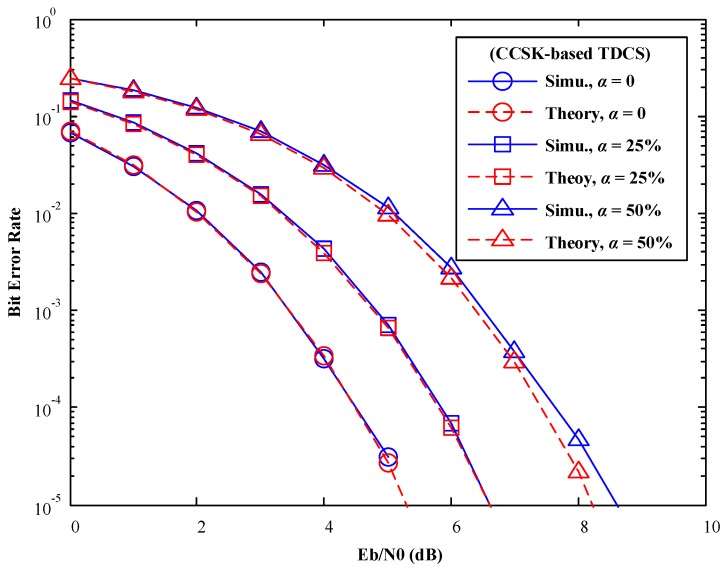
BER performance of TDCS-CCSK in AWGN channels (various mismatch factor α).

**Figure 8:**
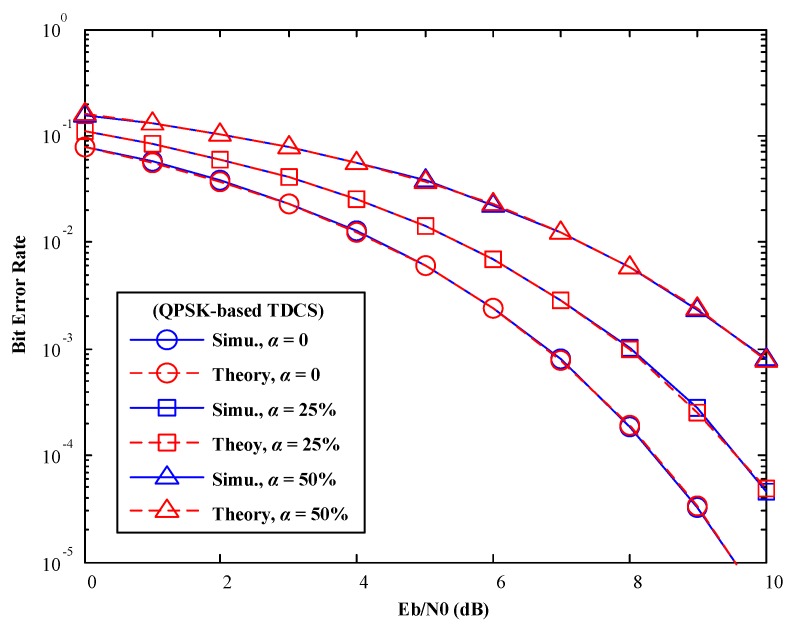
BER performance of TDCS-QPSK in AWGN channels (various mismatch factor α).

In Figure 7 and Figure 8, various mismatch factors are introduced to model various degree of SSM conditions. In spite of difference modulations methods in TDCS (CCSK or QPSK), Monte-Carlo simulation results match with our theoretical analysis perfectly. One observation is that, when the mismatch factor α increases, BER performance of the TDCS is deteriorated gradually, as both symbol detections suffer from the presence of SSM.

This deterioration seems to be slightly worse than expected when α=50% . This is because that the CCSK is a waveform modulation, in which symbol information relies on the PCF vector, and is recovered by detecting the tag of the max peak. As α increases, CCSK signaling no longer maintains its pseudo-orthogonal property, resulting in a severe rise of the side lobes in the PCF vector. In this way, Equation (11) serves as upper bound rather than an accurate result. 

### 4.2. Multipath Rayleigh Fading Channels

As mentioned above, BER performance for the TDCS in multipath fading channels is closely dependent on the channel conditions. Thus, it is assumed that the channel information is perfectly known to the receiver.

**Figure 9:**
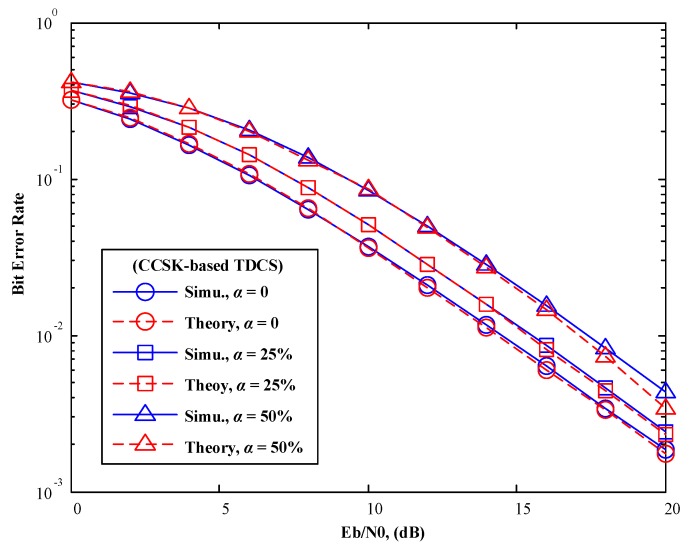
BER performance of TDCS-CCSK in multi-path channels (various mismatch factor α).

**Figure 10:**
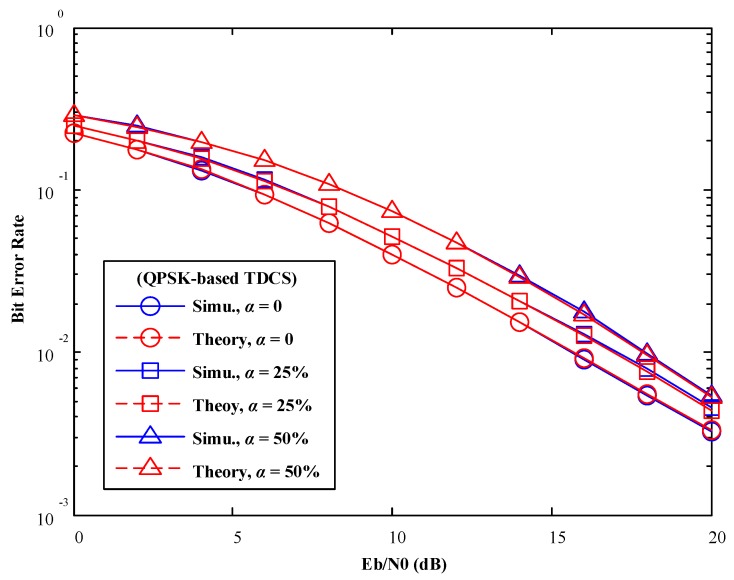
BER performance of TDCS-QPSK in multi-path channels (various mismatch factor α).

Meanwhile, a cyclic prefix with the length of 1/4 is used to combat inter-symbol interference and a one-tap zero-forcing frequency domain equalizer is considered. For Rayleigh channels, the general performance of both systems suffers from multipath fading even with ZF equalization, as shown in Figure 9 and Figure 10. However, this would not change the fact that each pair of simulations and theoretic lines still match rather well for a given α factor. This proves that the SSM influence has been accurately modeled in our semi-deterministic analysis (providing perfect channel estimation). Meanwhile, in CCSK-based TDCS, the simulation line still tends to get slightly inferior as α gets larger, due to the destruction of orthogonality in the PCF vector.

**Figure 11:**
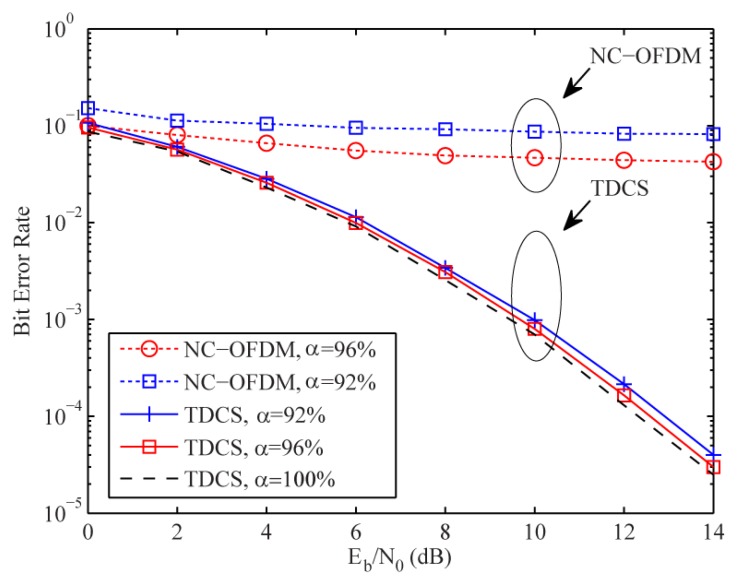
BER comparison of the TDCS and NC-OFDM.

Figure 11 shows the BER performances of TDCS for a={92%,96%} . We used a CR channel setting with four spectrum holes and 50% available subcarriers. For analysis, we study the $E_{b}/N_{0}$ loss (compared to that of the perfect spectrum sensing case with *a* = 100%) at BER = 10^−3^. One can see that the BER curves for a={92%,96%} are very close to that of the perfect spectrum sensing, with only 0.15 dB and 0.4 dB $E_{b}/N_{0}$ loss, respectively. As expected, the BER performance is degraded gradually as η decreases. In contrast, when non-continuous OFDM (NC-OFDM) is used, the BER curves under the same η settings show very high error floors at all $E_{b}/N_{0}$ levels, indicating that it is very sensitive to spectrum sensing mismatch. Therefore, our proposed TDCS system is more robust against modest spectrum sensing mismatch.

## 5. Conclusions

In this work, we discussed the TDCS system with respect to the IoT, where TDCS has proved to not only be a competitive candidate of traditional CR overlay technology, but also of great prospect in the IoT. However, due to some strict requirements, especially the need of ideal spectrum sensing results between transceivers, cognitive radio has limited its theoretical analysis and practical studies. This leads us to study the performance of TDCS in the presence of SSM. By adopting an intuitive spectral bin representation and by investigating QPSK and CCSK, it is found that the influence of SSM can be analyzed deterministically through a mismatch factor by applying it to the original SNR, regardless of the modulation methods. Finally, a general mathematical analysis for TDCS performance under any proposed mismatched spectrum sensing pattern is proposed, which may trigger some inspiration for further studies.

## Figures and Tables

**Table 1:** Simulation parameters.

Channels	AWGN Channel	COST207RAx6 Channel
Modulation model	CCSK/QPSK	CCSK/QPSK
Mismatch Factor (α)	0	0
	25%	25%
	50%	50%

## References

[1] Mitola J., Maguire G.Q. (1999). Cognitive radio: Making software radios more personal. IEEE Pers. Commun..

[2] Mitola J. (2000). Cognitive Radio: An Integrated Agent Architecture for Software Defined Radio. Ph.D. Thesis.

[3] He S., Huang Y., Jin S., Yang L. (2013). Coordinated beamforming for energy efficient transmission in multicell multiuser systems. IEEE Trans. Commun..

[4] Swackhammer P.J., Temple M.A., Raines R.A. Performance simulation of a transform domain communication system for multiple access applications. Proceedings of the IEEE Military Communications Conference Proceedings (MILCOM 1999).

[5] Nunez A.S., Temple M.A., Mills R.F., Raines R.A. Interference Avoidance in Spectrally Encoded Multiple Access Communications Using MPSK Modulation. Proceedings of the 2005 IEEE Wireless Communications and Networking Conference.

[6] Nunez A.S. (2012). Interference Suppression in Multiple Access Communications Using *M*-Ary Phase Shift Keying Generated Via Spectral Encoding. Master’s Thesis.

[7] Han C., Wang J., Yang Y., Li S. (2008). Addressing the control channel design problem: OFDM-based transform domain communication system in cognitive radio. Comput. Netw..

[8] Zhao Y., Anjum M.N., Song M., Xu X., Wang G. Optimal Resource Allocation for Delay Constrained Users in Self-Coexistence WRAN. Proceedings of the IEEE Global Communications Conference.

[9] Jiao Y., Yin P., Joe I. (2016). Clustering scheme for cooperative spectrum sensing in cognitive radio networks. IET Commun..

[10] Jiao Y., Joe I. (2016). Markov model-based energy efficiency spectrum sensing in Cognitive Radio Sensor Networks. J. Comput. Netw. Commun..

[11] Zhao Y., Hong Z., Wang G., Huang J. High-Order Hidden Bivariate Markov Model: A Novel Approach on Spectrum Prediction. Proceedings of the 25th IEEE International Conference on Computer Communication and Networks (ICCCN).

[12] Zhao Y., Pradhan J., Wang G., Huang J. Experimental approach: Two-stage spectrum sensing using GNU radio and USRP to detect primary user’s signal. Proceedings of the 31st Annual ACM Symposium on Applied Computing.

[13] So J., Sung W. (2016). Group-Based Multibit Cooperative Spectrum Sensing for Cognitive Radio Networks. IEEE Trans. Veh. Technol..

[14] Cacciapuoti A.S., Caleffi M., Izzo D., Paura L. (2011). Cooperative Spectrum Sensing Techniques with Temporal Dispersive Reporting Channels. IEEE Trans. Wirel. Commun..

[15] Cacciapuoti A.S., Caleffi M. (2015). Interference Analysis for Secondary Coexistence in TV White Space. IEEE Commun. Lett..

[16] Ding G., Wang J., Wu Q., Yao Y.D., Song F., Tsiftsis T.A. (2016). Cellular-Base-Station-Assisted Device-to-Device Communications in TV White Space. IEEE J. Sel. Areas Commun..

[17] Caleffi M., Cacciapuoti A.S. (2016). Optimal Database Access for TV White Space. IEEE Trans. Commun..

[18] Caleffi M., Cacciapuoti A.S. (2016). On the achievable throughput over TVWS sensor networks. Sensors.

[19] Cacciapuoti A.S., Caleffi M., Paura L. (2016). On the probabilistic deployment of smart grid networks in TV white space. Sensors.

[20] Lu Q., Yang S., Liu F. (2017). Wideband Spectrum Sensing Based on Riemannian Distance for Cognitive Radio Networks. Sensors.

[21] Han C., Wang J., Gong S., Li S. Detection and Performance of the OFDM-based Transform Domain Communication System. Proceedings of the 2006 IEEE International Conference on Communications, Circuits and Systems Proceedings.

[22] Hu S., Guan Y.L., Bi G., Li S. (2013). Spectrally efficient transform domain communication system with quadrature cyclic code shift keying. IET Commun..

[23] Hu S., Wu G., Xiao Y., Lei X., Li S. (2013). Design of Low PAPR Fundamental Modulation Waveform for Transform Domain Communication System. Wirel. Pers. Commun..

[24] Hu S., Wu G., Xiong W.H., Xiao Y., Dan L, Li S. TDCS Waveform Design for MUI-Free Cognitive Radio Networks. Proceedings of the IEEE 78th Vehicular Technology Conference (VTC Fall).

[25] Hu S., Liu Z., Guan Y.L., Xiong W.H., Li B. (2014). Sequence Design for Cognitive CDMA Communications under Arbitrary Spectrum Hole Constraint. IEEE J. Sel. Areas Commun..

[26] Dillard G.M., Reuter M., Zeidler J., Zeidler B. (2003). Cyclic code shift keying: A low probability of intercept communication technique. IEEE Trans. Aerosp. Electron. Syst. Soc..

[27] Fumat G., Charge P., Zoubir A., Fournier-Prunaret D. (2011). Transform domain communication systems from a multidimensional perspective impacts on bit error rate and spectrum efficiency. IET Commun..

[28] Chakravarthy V.D., Nunez A.S., Stephens J.P. (2005). TDCS, OFDM, and MC-CDMA: A Brief Tutorial. IEEE Radio Commun..

[29] Stevenson C.R., Chouinard G., Lei Z., Hu W., Shellhammer S.J., Caldwell W. (2009). IEEE 802.22: The first cognitive radio wireless regional area network standard. IEEE Commun. Mag..

[30] Papoulis A., Pillai S.U. (2002). Probability, Random Variables, and Stochastic Processes.

